# Supplementary material for: Diversity of edible insects in a Natural World Heritage Site of India: entomophagy attitudes and implications for food security in the region
Source: PeerJ. 2020 Nov 16;8:e10248. doi: 10.7717/peerj.10248 (PMC7676356; doi:10.7717/peerj.10248)
Supplement: Supplemental Information 2 [file peerj-08-10248-s002.pdf]

# Questionnaire for Data Collection on Edible Insects in Manas National Park, Assam

**Project: Quantitative and Qualitative Survey of Edible Insects in various National Parks of Assam, India**
**Session: 2018-2019**

This project has been approved by Office of the Principal Chief Conservator of Forests (Wildlife) and Chief Wildlife Warden, Government of Assam, India

Name of the interviewee: ______________________ Age: ________________ Sex: ____________
Address: Village__________________________ Revenue Block___________ District_____________
Religion (Put ✓): Hinduism/Islam/Christian/Others Caste: ______________________________
Community (Put ✓): Rabha/Sarania/Adivashi/Bodo/Others

Brief outline of edible insects:

Reason of eating:

| Sl. No. | Species Name/ Local Name | Month of availability | Collection site(s)* | Edible parts** | Mode of eating*** | Medicinal value if any | Misc. |
|---|---|---|---|---|---|---|---|
| 1. | | | | | | | |
| 2. | | | | | | | |
| 3. | | | | | | | |
| 4. | | | | | | | |
| 5. | | | | | | | |
| 6. | | | | | | | |
| 7. | | | | | | | |
| 8. | | | | | | | |
| 9. | | | | | | | |
| 10. | | | | | | | |
| 11. | | | | | | | |
| 12. | | | | | | | |
| 13. | | | | | | | |
| 14. | | | | | | | |
| 15. | | | | | | | |
| 16. | | | | | | | |
| 17. | | | | | | | |
| 18. | | | | | | | |
| 19. | | | | | | | |
| 20. | | | | | | | |

*Collection sites: Trees/Grasses/Crop field/forces/river/pond/swampy areas/backyard forest
**Eidible parts: Whole bpdy/Larvae/Pupae/Eggs
*** Mode of eating: Fried/curry/smoked/raw/roasted/paste

Number of total members in the family: ____________________________

| Above 60 years | Between 4-60 years | Between 20-40 years | Below 20 years |
|---|---|---|---|
| | | | |

Number of insect consumers in the family: ____________________________

| Above 60 years | Between 4-60 years | Between 20-40 years | Below 20 years |
|---|---|---|---|
| | | | |

If collected for sale:

| Name of the market(s) | How frequently collected for sale (Put ✓) | Amount/quantity of insect selling (per month) | Site of collection | Price (in Rs.) |
|---|---|---|---|---|
| | Daily/weekly/monthly | | | |

Signature of Interviewee

Signature of Researcher
